# Differences among six woody perennials native to Northern Europe in their level of genetic differentiation and adaptive potential at fine local scale

**DOI:** 10.1002/ece3.3824

**Published:** 2018-01-25

**Authors:** Albin Lobo, Jon Kehlet Hansen, Lars Nørgaard Hansen, Erik Dahl Kjær

**Affiliations:** ^1^ Department of Geosciences and Natural Resource Management (IGN) University of Copenhagen Frederiksberg C Denmark

**Keywords:** climate change, life‐history traits, local adaptation, phenology, quantitative genetics

## Abstract

The ability of perennial species to adapt their phenology to present and future temperature conditions is important for their ability to retain high fitness compared to other competing plant species, pests, and pathogens. Many transplanting studies with forest tree species have previously reported substantial genetic differentiation among populations within their native range. However, the question of “how local is local” is still highly debated in conservation biology because studies on genetic patterns of variation within and among populations at the local scale are limited and scattered. In this study, we compare the level of genetic differentiation among populations of six different perennial plant species based on their variation in spring flushing. We assess the level of additive genetic variation present within the local population. For all six species, we find significant differentiation among populations from sites with mean annual temperature ranging between 7.4°C and 8.4°C. The observed variation can only be partly explained by the climate at the site of origin. Most clear relationship between early flushing and higher average spring temperature is observed for the three wind‐pollinated species in the study, while the relations are much less clear for the three insect‐pollinated species. This supports that pollination system can influence the balance between genetic drift and natural selection and thereby influence the level of local adaptation in long‐lived species. On the positive side, we find that the native populations of woody plant species have maintained high levels of additive genetic variation in spring phenology, although this also differs substantially among the six studied species.

## 1 INTRODUCTION

Phenology is important for fitness of perennial species. Higher spring temperatures are expected to prolong the growing seasons due to earlier bud burst (Dragoni et al., 2011; Menzel & Fabian, 1999; Menzel et al., 2006; Richardson et al., 2010, 2013; Vitasse, Delzon, Dufrêne, et al., 2009), while lack of synchrony between phenology and occurrence of spring frost events increases risk of damage to early flushing plants (Duputié, Rutschmann, Ronce, & Chuine, 2015). Yet again, raised temperatures with no frost events and changes in daily temperature fluctuations could also result in later bud burst and influence time between bud development and growth cessation (Chmura et al., 2011; Rohde, Bastien, & Boerjan, 2011; Way, 2011). Several studies have documented substantial variation among populations in their phenology reflecting their geographic origin (Alberto, Derory, et al., 2013; Chuine & Beaubien, 2001; Salmela, Cavers, Cottrell, Iason, & Ennos, 2013). It is therefore fair to assume that genetic variation within species combined with divergent selection has played an important role for the ability of many tree species to thrive in a large distribution range across strong environmental gradients (Brousseau et al., 2016; Kawecki & Ebert, 2004; Pluess et al., 2016). However, species differ in their response and spring and autumn phenology may show different patterns. A study based on 59 tree species from similar climatic clines thus showed a relative clear pattern with respect to bud set in autumn but less clear pattern in spring bud burst (Alberto, Aitken, et al., 2013). The expectation of local adaptation often leads restoration and conservation programs to focus on gene pools at the local scale rather than the regional scale (Stanturf et al., 2015), but only few studies have actually assessed the variation at the local scale (within ~100 km).

Fast climate change calls for high phenotypic plasticity (Valladares et al., 2014) and genetic variation in relevant traits in plant populations (Franks, Weber, & Aitken, 2014) in order to ensure continued adaptation. Many tree species have revealed phenotypic plasticity and demonstrated their ability to thrive and reproduce when transferred to new areas (Kjær, Lobo, & Myking, 2014; Lascoux, Glémin, & Savolainen, 2016; Myking, Rusanen, Steffenrem, Kjær, & Jansson, 2016; Rehfeldt, Ying, Spittlehouse, & Hamilton, 1999). However, there are indications of declining plasticity in phenology in spring with continued global warming (Fu et al., 2015) and increased frequency of warm periods in the early spring may lead to frost backlashes (Jönsson & Bärring, 2011). The temperatures are likely to change in the future, but the seasonal variations in day length remain the same. Species will therefore respond differently as regards phenology to temperature changes depending on the species‐specific importance of day length versus heat sum in controlling flushing time (Alberto, Aitken, et al., 2013; Caffarra & Donnelly, 2011; Fu et al., 2015; Vitasse, Delzon, Bresson, Michalet, & Kremer, 2009; Vitasse, Delzon, Dufrêne, et al., 2009).

Variation among individual genotypes in their phenology triggers selection at the population level in favor of the new conditions, if the variation is in fitness traits and expressed with moderate or high heritability (Alberto, Aitken, et al., 2013; Falconer & Mackay, 1996). Genetic differentiation within and among population is therefore important for the species ability to adapt to climate change (Kubisch, Holt, Poethke, & Fronhofer, 2014). However, natural selection is not the only evolutionary force, because the combined actions of natural selection, gene flow and genetic drift drive the level of genetic variation within and among populations in fitness traits (Nadeau, Meirmans, Aitken, Ritland, & Isabel, 2016). The realized patterns therefore depend on effective population sizes, force of natural selection and limitations in pollen and seed dispersal (Aitken & Whitlock, 2013; Savolainen, Pyhäjärvi, & Knürr, 2007). Natural selection tends to increase the level of genetic variation among populations, but reduce the variation within populations leading to local adaptation. Fragmentation into small populations can create drift that also increase variation among population variation, but without supporting local adaptation. Small populations can rather reduce fitness due to inbreeding, at least in outcrossing species. Pollination and seed dispersal across the landscape among populations will counteract the fragmentation, but seed and pollen dispersal across environmental gradients will also counteract natural selection and thereby reduce the ability of populations to adapt to specific local conditions (Kubisch et al., 2014). The level of gene flow is expected to differ among woody species depending on occurrence, landscape features, mating system, pollen, and seed dispersal vectors. We therefore expect that the degree of local differentiation—and the degree to which this reflect local adaptation—will differ among woody plant species depending on their major life‐history traits.

### 1.1 Objectives of this study

On the above background, the objectives of this study were to use data on spring phenology to

1. Assess the degree to which woody perennials reveal fine‐scale genetic differences among geographically and climatic close (differences in mean annual temperature around 1°C) populations, and test whether differences (if any) influence the fitness revealing signs of local adaptation.
2. Estimate levels of genetic variation within and among populations in spring phenology and compare the levels of population differentiation (*Q*_ST_) and additive genetic variation (*V*_A_) among species.

Based on the results, we discuss the implications for dynamic conservation of genetic resources and the ability of woody plant species to adapt to future climate conditions.

## 2 MATERIALS AND METHODS

**Figure 1:**
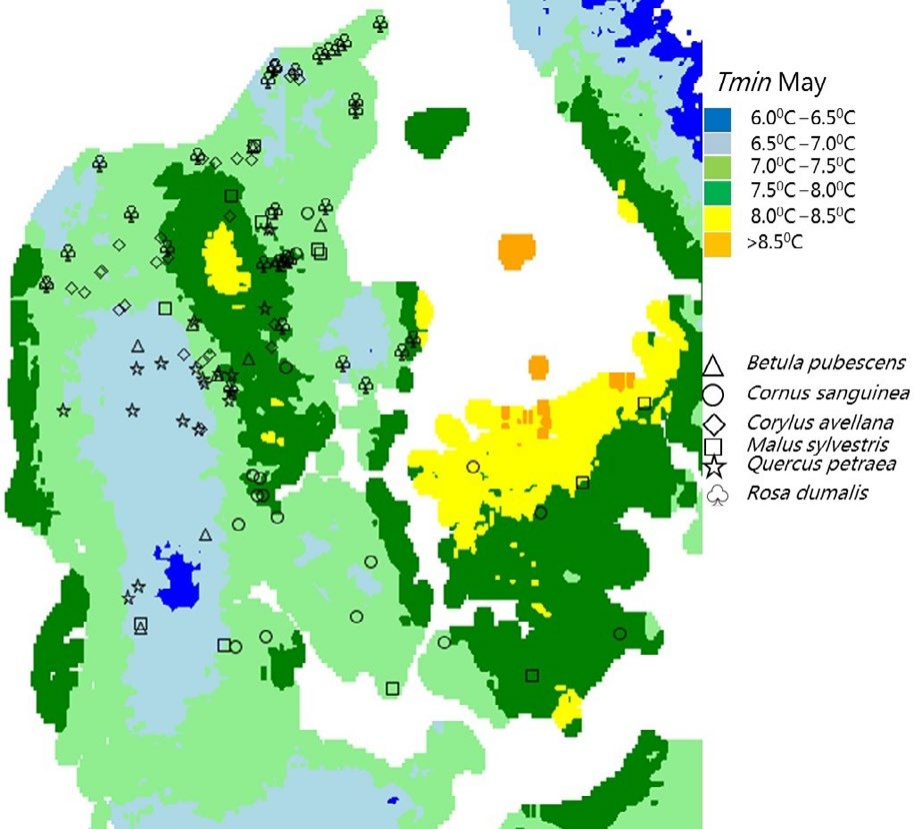
Locations of original populations for the different species in the study are spread across different climate zones (here represented by temperature minimum in May [*T*_min_])

Six broadleaved species in Denmark were studied, of which three are insect pollinated—Common dogwood (*Cornus sanguinea* L.), European Crab Apple (*Malus sylvestris* (L.) Mill.) and Glaucous Dog Rose (*Rosa dumalis* (Bechst) Boulay), while three are wind pollinated—Downy birch (*Betula pubescens* Ehrh.), Common Hazel (*Corylus avellana* L.) and Sessile oak (*Quercus petraea* (Matt.) Liebl.). We used data from progeny trials with offspring from open pollinated trees based on seed collected from putative natural populations across Denmark (Figure 1). Description of the field trials, location of populations, and climate information at the population site and information on families within each population are provided in Appendix S1.

### 2.1 Assessment of phenotypic data and estimation of variance components

We used spring phenology assessed as bud burst scores using a scale from 0 to 8, where class 0 was closed winter buds and class 8 was fully opened leaves. The time of assessment was adapted for each species so that the trees in the trials were approximately in the middle of their flushing time (average values of bud burst score between 3 and 5). This was to ensure maximum variation among individuals in their bud burst ranking. Bud burst for *B. pubescens, C. sanguinea, M. sylvestris,* and *R. dumalis* were assessed in spring 2010, while assessments of *C. avellana* and *Q. petraea* were from spring 2013. Height was measured in the year of phenology assessment except for *R. dumalis*.

All analyzes were made per species, because they were tested in different common garden trials. The variation among trees in their phenological stage was separated into variance and covariance components reflecting genetic differences among and within populations as implemented in the software ASReml (Gilmour, Gogel, Cullis, & Thompson, 2009) from the model described below.

Population values were predicted by application of mixed model (1), and genetic variance component corresponding to each of the random effects in the model was estimated from the analysis;

(1) $$Y_{\mathrm{ijkl}}=\mu +B_{i}+P_{j}+\lambda_{\mathrm{ij}}+f_{k(j)}+\tau_{\mathrm{ikj}}+\varepsilon_{\mathrm{ijkl}}$$

where *Y*_*ijkl*_ is the trait measured for tree *l*, μ is the overall mean of the trait, *B*_*i*_ is the fixed block effect, *P*_*j*_ is the fixed population effect, λ_*ij*_ is the fixed population by block interaction, *f*_*k*(*j*)_ is the random effect of family within population, τ_*ijk*_ is the random effect of plot *k*, and ε_*ijkl*_ is the residual. *Q*_ST_ values were estimated from model (1) as well having population effects as random.

The significance of the genetic variances of traits within sites and genotype by environment interaction across sites was tested using the log likelihood ratio (Gilmour et al., 2009). The significance of the populations was tested using the Satterthwaite approximation (Satterthwaite, 1946) in the procedure GLM in the statistical software program SAS (SAS Institute Inc. 2011).

### 2.2 Quantitative genetic analysis

We estimated the additive genetic variance (*V*_A_) as 4σ_f_^2^ and narrow sense heritability within sites as; ⌢h2=4σf2/(σf2+στ2+σε2), where σ_f_^2^ is the estimated family variance, σ_τ_^2^ is the estimated plot variance, and σ_ε_^2^ is the estimated within plot variance. The phenotypic variance was estimated as ⌢VP=σf2+στ2+σε2. Families were considered to represent groups of half‐sibs. This assumption will overestimate the additive genetic variance, heritability, and expected response to selection, if the offspring within progeny groups on average are more related than half‐sibs (Falconer & Mackay, 1996). While the assumption of half‐sibs may provide a good fit for most trees species (Kjær, McKinney, Nielsen, Hansen, & Hansen, 2012; Larsen & Kjær, 2009), the situation may be more complicated for *R. dumalis,* where polyploidy may be present as the variance between families is biased due to a fraction of the dominance genetic variance, even if the families consist of pure half‐sibs (Roberts, Gladis, & Brumme, 2009).

The degree of differentiation among populations was estimated as the *Q*_ST_ values (Spitze, 1993) using the formula;

⌢QST=⌢VPOP/(⌢VPOP+2⌢VA)

where ⌢VPOP is the estimated variance between populations, and ⌢VA is the estimated additive genetic variance.

Variance components, narrow sense heritability estimates, *Q*_ST_ estimates, and their standard errors were estimated from model (1) using the software ASReml (Gilmour et al., 2009).

### 2.3 Support to the hypothesis of location adaptation

Climate data for the locations of origins were estimated with the ClimateEU v4.63 software package following the methodology described by (Hamann, Wang, Spittlehouse, & Murdock, 2013). Weighted regression analysis to test relationship between phenology of populations and climate at the population sites was carried out using procedure REG in SAS (SAS Institute Inc. 2011) having the predicted values of populations as dependent variable and climate variables as explanatory variables. One divided by the error variance for the predicted population values were used as weights. The climate variables tested for bud burst were monthly minimum temperatures and differences between monthly maximum and minimum temperatures in March, April, May and June, because these data are expected to provide good proxies for the risk of frost occurring after flushing. A backward selection approach (5% level) was used for the selection of climate variables that could best explain the variation in flushing time. The relationship between geographic distances and difference in average budburst was tested by a Mantel test as implemented in R version 3.2.2 (Dray & Dufour, 2007).

### 2.4 Fitness effects of spring phenology

Genetic correlations between bud burst and height growth were estimated based on individual tree data to assess whether the height as a proxy for realized fitness in the present climate varied with the phenology. Genetic correlations could be estimated between survival and phenology based on plot means, because the plants were grown in family plots in all trials (see Appendix S1). Genetic correlations were estimated according to Falconer and Mackay (1996) as ⌢rg=σfxyσfx2σfy2 , where σ_*fxy*_ is the family (within population) covariance between trait *x* and *y*, σ_*fx*_^2^ is the family (within population) variance for trait *x*, and σ_*fy*_^2^ is the family (within population) variance for trait *y*.

## 3 RESULTS

### 3.1 Presence of genetic variation within and among populations in bud burst

**Figure 2:**
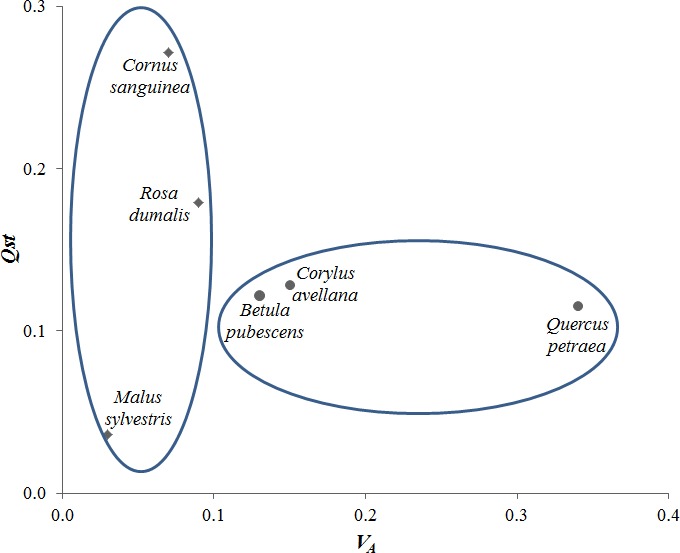
Difference among species in genetic variation within populations (*V*_A_ = additive genetic variance) and population differentiation (*Q*_ST_ value) for bud 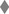 burst insect‐pollinated species, 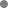 wind‐pollinated species

Genetic variation both within and among populations was significant in all six species, but with large differences among the species. The additive genetic variance for bud burst (*V*_A_) thus ranged from 0.07 in *C. sanguinea* to 0.34 in *Q. petraea* (Table 1), while the level of differentiation among populations (*Q*_ST_) ranged from 0.04 in *M. sylvestris* to 0.27 in *C. sanguinea* (Table 2). The three wind‐pollinated species *B. pubescens, C. avellana* and *Q. petraea* showed the highest level of additive variance, while the insect‐pollinated species *C. sanguinea* and *R. dumalis* showed the highest level of population differentiation as measured by the *Q*_ST_ (Figure 2).

**Table 1:** Family variation and genetic parameters for bud burst in different species

Species	Trait	Mean	*p*‐Value family	*V*_A_	*V*_P_	*h* ^2^	*SE*
*Betula pubescens*	Bud burst 24 April 2010	3.27	.003	0.15	0.30	0.50	0.12
*Cornus sanguinea*	Bud burst 6 May 2010	3.81	<.0001	0.07	0.27	0.24	0.11
*Corylus avellana*	Budburst 24 April 2013	3.09	<.0001	0.13	0.27	0.47	0.09
*Malus sylvestris*	Bud burst 28 April 2010	4.05	<.0001	0.03	0.10	0.31	0.08
*Quercus petraea*	Bud burst 24 May 2013	4.64	<.0001	0.34	0.58	0.58	0.26
*Rosa dumalis*	Bud burst 26 April 2010	3.58	<.0001	0.09	0.21	0.41	0.14

*V*_A_ = additive genetic variance, *V*_P_ = *p* variance, *h* ^2^ = narrow sense heritability, *SE* = standard error for *h* ^2^.

**Table 2:** Level of population differentiation for bud burst in different species

Species	Trait	*Q*_st_	*SE*	*p*‐Value population	Max. pop. diff.^a^
*Betula pubescens*	Bud burst 24 April 2010	0.13	0.08	<.001	0.71
*Cornus sanguinea*	Bud burst 6 May 2010	0.27	*0.14*	<.001	0.88
*Corylus avellana*	Budburst 24 April 2013	*0.12*	*0.05*	<.0001	0.86
*Malus sylvestris*	Bud burst 28 April 2010	0.04	*0.03*	.02	0.28
*Quercus petraea*	Bud burst 24 May 2013	0.13	0.09	.0003	2.45
*Rosa dumalis*	Bud burst 26 April 2010	0.18	0.09	<.001	1.52

a Maximum difference between populations in bud burst score.

QST = quantitative genetic differentiation among populations in bud burst, SE = standard error for QST

### 3.2 Relationship of phenology with growth, fitness, distance between populations and climate at original population site

**Figure 3:**
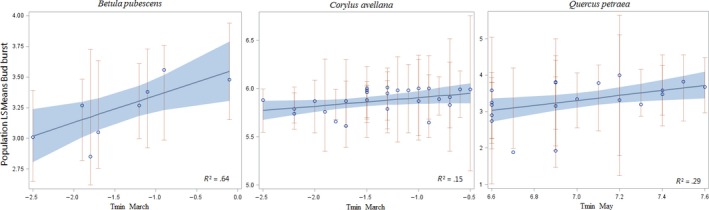
Weighted regression between population means of bud burst score and *T*_min_ (temperature minimum) of different months in spring at original population site in different species

**Figure 4:**
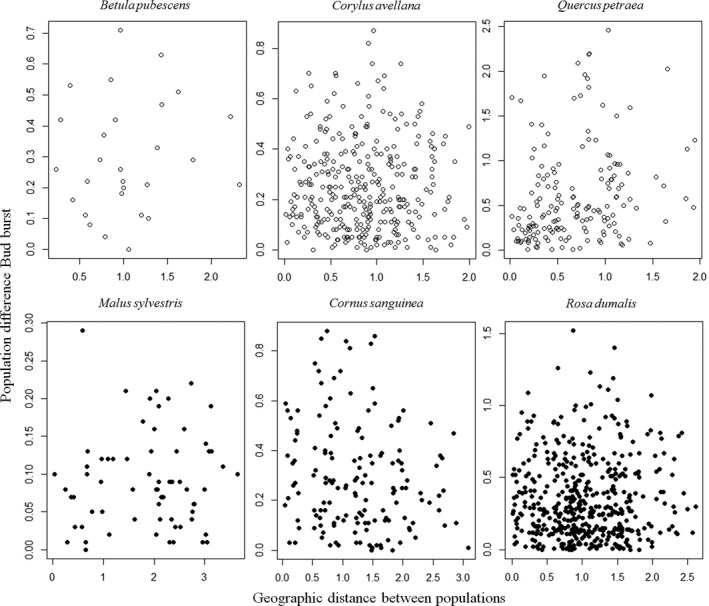
Population genetic differentiation in bud burst show no relationship with geographic distance (in 100 km) between them in different species

The regression between bud burst of populations and population site minimum temperatures in March were significant (*p < .05*), or close to significant (*p = .05*) for *B. pubescens* and *C. avellana* (Figure 3), and the regression between bud burst of populations and population site minimum temperatures in May was close to significant (*p = .05*) for *Q. petraea* (Figure 3). This corresponds to the general time for bud burst, where *B. pubescens* and *C. avellana* flush before *Q. petraea* in Denmark and indicate that spring frost event has played an important role in developing the pattern of variation among population in these species. Similar significant correlations were not observed in any of the three insect‐pollinated species. Mantel tests for correlation between geographic distance and difference in bud burst at population were nonsignificant in all the species (Figure 4). Survival was not significantly correlated to bud burst data in any of the species evaluated, and a significant additive genetic correlation between height and budburst was only observed in *B. pubescens (rg = *0.35; *SE 0.23*).

## 4 DISCUSSION

Our results show that fine‐scale local genetic differentiation in fitness traits such as phenology indeed can be present and to a larger extent than previously anticipated in the Danish gene conservation strategy (Graudal, Kjær, & Canger, 1995). However, the analysis across the six different species did not provide unique patterns of local adaptation, as the absence of significant regression between bud burst time and spring temperature in the three insect‐pollinated species suggests that the variation among population is not always simply reflecting local adaptation. It is rather more likely a result of natural selection and neutral processes simultaneously acting as predicted by theory (Nadeau et al., 2016).The fact that populations in all studied six species were significantly differentiated in their spring phenology suggests that local populations of woody perennials more often than not are genetically differentiated, and this can be the case even if they only are separated by 10 to 35 km (Figure 4) and growing in areas with low variation in altitude and where the spring temperature varies only between 1°C and 2.5°C (Figure 3). This finding should be compared to the prediction of Northern Europe becoming 2–4°C warmer by the year 2100 (Nikulin, Kjellström, Hansson, Strandberg, & Ullerstig, 2011). However, the observed high level of additive genetic variation in the studied species indicates that the native populations of woody species do possess substantial evolutionary potential. Divergent natural selection is possible within few generations, if the timing of phenology creates large differences in fitness. This is supported by the fact that the observed patterns in our study for *B. pubescens*,* C. avellana,* and *Q. petraea* (Figure 3) are at least partly due to past natural selection in favor of local adaption.

The presence of local adaptation and evolutionary potential in woody species are often attributed to their life‐history traits including pollination mechanisms and associated genetic processes, and it is therefore interesting that the patterns were different for the wind‐ and insect‐pollinated species in our study. The result that the three wind‐pollinated, continuous distributed species (*B. pubescens, C. avellana, Q. petraea)* were characterized by having high levels of genetic variation within populations (right circle of Figure 2) supports the general assumption that wind‐pollinated, widely distributed species have allocated genetic variation within populations to a larger extent than insect‐pollinated species with small and scattered populations (Petit & Hampe, 2006; Smith & Donoghue, 2008). The results correspond to the expectation that long‐distance gene flow through wind pollination will maintain connectivity among populations (lowering *Q*_ST_) and counteract loss of genetic variation (maintaining high *h* ^2^) within populations (Sork, 2016). High levels of gene flow among populations across landscapes have been reported in the wind‐pollinated *Quercus* (Gerber et al., 2014). The insect‐pollinated *C. sanguinea* was in the opposite corner of *Q. petraea* in Figure 2, reflecting a much higher proportion of genetic variation located among populations. *C. sanguinea* in general occurs in scattered and small populations in Denmark (Ødum, 1968). Seed dispersal by birds (Krüsi & Debussche, 1988) may generate long‐distance gene flow across the landscape, but the observed level and distribution of genetic diversity in the present study point toward genetic drift rather than natural selection as important driver behind the observed population differentiation. The true difference between *Q. petraea* and *C. sanguinea* in level of genetic variation may be even higher, because the assumption of half‐sib relationship within seed from single tree collection is likely to create a bias toward overestimation of *V*_A_ in the rose as discussed in M&M above. *M. sylvestris* is again a different case in Figure 2, with low level of genetic differentiation but also relatively low additive genetic variance. Pollinating insects have been reported to visit primarily trees in immediate vicinity, but long‐distance pollination events are also likely to occur (Larsen & Kjær, 2009; Reim et al., 2015) and seeds moved by birds and deer that feed on the wild apples also create long‐distance gene flow (Larsen & Kjær, 2009). Studies based on neutral SSR markers revealed low level of population differences within Denmark (*F*_ST_ = 0.03) (Larsen, Asmussen, Coart, Olrik, & Kjær, 2006), which is very close to the estimated *Q*_ST_ = 0.04 for flushing time in the present. Therefore, the observed variation among populations in *M. sylvestris* may have been generated by random processes without significant effects of divergent selection for flushing time.

The studied populations have in general maintained a high level of genetic variation and thereby possess a high ability to adapt to new conditions if exposed to natural selection in favor of—or against—early flushing. The question is how fast species can adapt under the anticipated rapid increase in temperature over the next 100 years, and how important such changes are for the fitness of the species. In *B. pubescens*, the maximum difference in minimum temperature in March of the populations tested in this study was 2.4°C (Figure 3). Even with a conservative estimate of a generation time of 50 years (Hynynen et al., 2010), the results suggest that *B. pubescens* possesses the resilience at the population level to change phenology corresponding to the predicted increase in temperature. A similar conclusion can probably be drawn for *Q. petraea* also (Figure 3). For many of the other studied species, the generation time is possibly less than 20 years (e.g., *R. dumalis*,* M. sylvestris*,* C. avellana,* and *C. sanguinea*), and for these species, it therefore also seems reasonable to assume that populations can evolve in spring phenology with a speed that can match the predicted increase in temperature. The response will only be realized if early bud burst has substantial influence on fitness (survival and reproduction) or because of directional selection implemented in domestication programmes. The estimates in the present study refer to the latter situation, because the heritability was estimated in managed progeny test and the realized heritability in natural population may be substantially lower due to higher environmental and developmental heterogeneity. In Denmark, all major woody plant species are included in domestication programs based on breeding seed orchards in order to support effective and rapid selection for continued fitness (Kjær et al., 2009).

Our finding on genetic differences among geographically close populations (cf. Table 2) supports that selectively divergent phenotypes can be maintained even if there are options for gene flow among these populations as suggested by Aitken and Bemmels (2016). Kremer et al. (2012) argue that the positive effect of gene flow on adaptation of trees to climate change is larger than the negative effect due to the introduction of genetic variation in adaptive traits. Species are different with respect to their genetic differentiation within and among their populations as revealed in our study. Assisted migration for genetic enrichment can serve as a supplement to genetic management based on native populations; especially, the species associated with small population sizes and limited gene flow. Populations at the low latitudinal limit of the species range have in general maintained high biological diversity over time as shown for, for example, *Abies alba* (Bergmann, Gregorius, & Larsen, 1990; Larsen, 1986) and southern populations should therefore be considered as a source of gene pool for assisted migration of genotypes under the future climate predictions (Hampe & Petit, 2005). The populations in the present study are close to the northern distribution range of the species (except for *B. pubescens*) and genetic diversity may therefore be lower compared to populations closer to the refugial areas. But we still observed substantial level of genetic variation in the fitness‐related trait. For species occurring in extremely small fragmented populations assisted migration at a more localized landscape level may still be desirable to counteract inbreeding within these populations due to random drift. Adaptive potential of a species is not only determined by the presence of genetic variation within and among populations as studied here, but also by the presence of phenotypic plasticity in adaptive traits (Nicotra et al., 2010). Final conclusions on the adaptive potential of species to climate change will therefore also depend on the role of plasticity in local adaptation in the studied species.

## DATA ACCESSIBILITY

Data for this study is available at University of Copenhagen—Electronic Research Data Archive (UCPH‐ERDA).

## CONFLICT OF INTEREST

None declared.

## AUTHOR CONTRIBUTION

Albin Lobo was responsible for the design of study, analysis and interpretation of data, and writing of the manuscript. Jon Kehlet Hansen was responsible for supervision of the work, design of study, and writing and approving of manuscript. Lars Nørgaard Hansen was responsible for field data collection, and revising and approving the final version of the manuscript before submission. Erik Dahl Kjær was responsible for overall supervision of the study, conception and design of work, data interpretation, and writing and final approval of the manuscript.

## References

[ece33824-bib-0001] Aitken, S. N. , & Bemmels, J. B. (2016). Time to get moving: assisted gene flow of forest trees. Evolutionary Applications, 9, 271–290. https://doi.org/10.1111/eva.12293 27087852 10.1111/eva.12293 PMC4780373

[ece33824-bib-0002] Aitken, S. N. , & Whitlock, M. C. (2013). Assisted gene flow to facilitate local adaptation to climate change. Annual Review of Ecology Evolution and Systematics, 44, 367–388. https://doi.org/10.1146/annurev-ecolsys-110512-135747

[ece33824-bib-0003] Alberto, F. J. , Aitken, S. N. , Alía, R. , González‐Martínez, S. C. , Hänninen, H. , Kremer, A. , … Savolainen, O. (2013). Potential for evolutionary responses to climate change – Evidence from tree populations. Global Change Biology, 19, 1645–1661. https://doi.org/10.1111/gcb.12181 23505261 10.1111/gcb.12181 PMC3664019

[ece33824-bib-0004] Alberto, F. J. , Derory, J. , Boury, C. , Frigerio, J. M. , Zimmermann, N. E. , & Kremer, A. (2013). Imprints of natural selection along environmental gradients in phenology‐related genes of *Quercus petraea* . Genetics, 195, 495 https://doi.org/10.1534/genetics.113.153783 23934884 10.1534/genetics.113.153783 PMC3781976

[ece33824-bib-0005] Bergmann, F. , Gregorius, H.‐R. , & Larsen, J. B. (1990). Levels of genetic variation in European silver fir (*Abies alba*). Genetica, 82, 1–10. https://doi.org/10.1007/BF00057667

[ece33824-bib-0006] Brousseau, L. , Postolache, D. , Lascoux, M. , Drouzas, A. D. , Källman, T. , Leonarduzzi, C. , … Vendramin, G. G. (2016). Local adaptation in European Firs assessed through extensive sampling across altitudinal gradients in Southern Europe. PLoS ONE, 11, e0158216 https://doi.org/10.1371/journal.pone.0158216 27392065 10.1371/journal.pone.0158216 PMC4938419

[ece33824-bib-0007] Caffarra, A. , & Donnelly, A. (2011). The ecological significance of phenology in four different tree species: Effects of light and temperature on bud burst. International Journal of Biometeorology, 55, 711–721. https://doi.org/10.1007/s00484-010-0386-1 21113629 10.1007/s00484-010-0386-1

[ece33824-bib-0008] Chmura, D. J. , Anderson, P. D. , Howe, G. T. , Harrington, C. A. , Halofsky, J. E. , Peterson, D. L. , … Brad, S. (2011). Forest responses to climate change in the northwestern United States: Ecophysiological foundations for adaptive management. Forest Ecology and Management, 261, 1121–1142. https://doi.org/10.1016/j.foreco.2010.12.040

[ece33824-bib-0009] Chuine, I. , & Beaubien, E. G. (2001). Phenology is a major determinant of tree species range. Ecology Letters, 4, 500–510. https://doi.org/10.1046/j.1461-0248.2001.00261.x

[ece33824-bib-0010] Dragoni, D. , Schmid, H. , Wayson, C. , Potter, H. , Grimmond, C. , & Randolph, J. (2011). Evidence of increased net ecosystem productivity associated with a longer vegetated season in a deciduous forest in south‐central Indiana, USA. Global Change Biology, 17, 886–897. https://doi.org/10.1111/j.1365-2486.2010.02281.x

[ece33824-bib-0011] Dray, S. P. , & Dufour, A. B. A. (2007). The ade4 package: Implementing the duality diagram for ecologists. Journal of Statistical Software, 22, 1–20.

[ece33824-bib-0012] Duputié, A. , Rutschmann, A. , Ronce, O. , & Chuine, I. (2015). Phenological plasticity will not help all species adapt to climate change. Global Change Biology, 21, 3062–3073. https://doi.org/10.1111/gcb.12914 25752508 10.1111/gcb.12914

[ece33824-bib-0013] Falconer, D. S. , & Mackay, T. F. C. (1996). Introduction to quantitative genetics, 4th ed. Essex, UK: Longmans Green.

[ece33824-bib-0014] Franks, S. J. , Weber, J. J. , & Aitken, S. N. (2014). Evolutionary and plastic responses to climate change in terrestrial plant populations. Evolutionary Applications, 7, 123–139. https://doi.org/10.1111/eva.12112 24454552 10.1111/eva.12112 PMC3894902

[ece33824-bib-0015] Fu, Y. H. , Zhao, H. , Piao, S. , Peaucelle, M. , Peng, S. , Zhou, G. , … Janssens, I. A. (2015). Declining global warming effects on the phenology of spring leaf unfolding. Nature, 526, 104–107. https://doi.org/10.1038/nature15402 26416746 10.1038/nature15402

[ece33824-bib-0016] Gerber, S. , Gugerli, F. , Lascoux, M. , Buiteveld, J. , Cottrell, J. , Dounavi, A. , … Kremer, A. (2014). High rates of gene flow by pollen and seed in oak populations across Europe. PLoS ONE, 9, e85130 https://doi.org/10.1371/journal.pone.0085130 24454802 10.1371/journal.pone.0085130 PMC3890301

[ece33824-bib-0017] Gilmour, A. R. , Gogel, B. J. , Cullis, B. R. , & Thompson, R. (2009). ASReml user guide release 3.0. Hemel Hempstead: VSN International Ltd Retrieved from www.vsni.co.uk

[ece33824-bib-0018] Graudal, L. , Kjær, E. D. , & Canger, S. (1995). A systematic approach to the conservation of genetic resources of trees and shrubs in Denmark. Forest Ecology and Management, 73, 117–134. https://doi.org/10.1016/0378-1127(94)03497-K

[ece33824-bib-0019] Hamann, A. , Wang, T. , Spittlehouse, D. L. , & Murdock, T. Q. (2013). A comprehensive, high‐resolution database of historical and projected climate surfaces for western North America. Bulletin of the American Meteorological Society, 94, 1307–1309. https://doi.org/10.1175/BAMS-D-12-00145.1

[ece33824-bib-0020] Hampe, A. , & Petit, R. M. J. (2005). Conserving biodiversity under climate change: The rear edge matters. Ecology Letters, 8, 461–467. https://doi.org/10.1111/j.1461-0248.2005.00739.x 21352449 10.1111/j.1461-0248.2005.00739.x

[ece33824-bib-0021] Hynynen, J. , Niemistö, P. , Viherä‐Aarnio, A. , Brunner, A. , Hein, S. , & Velling, P. (2010). Silviculture of birch (*Betula pendula* Roth and *Betula pubescens* Ehrh.) in northern Europe. Forestry, 83, 103–119. https://doi.org/10.1093/forestry/cpp035

[ece33824-bib-0022] Jönsson, A. M. , & Bärring, L. (2011). Ensemble analysis of frost damage on vegetation caused by spring backlashes in a warmer Europe. Natural Hazards and Earth Systems Sciences, 11, 401–418. https://doi.org/10.5194/nhess-11-401-2011

[ece33824-bib-0023] Kawecki, T. J. , & Ebert, D. (2004). Conceptual issues in local adaptation. Ecology Letters, 7, 1225–1241. https://doi.org/10.1111/j.1461-0248.2004.00684.x

[ece33824-bib-0024] Kjær, E. D. , Hansen, L. N. , Graudal, L. O. V. , Olrik, D. C. , Ditlevsen, B. , Jensen, V. , & Jensen, J. S. (2009). The Danish programme for domestication of native woody species. Genetic conservation and management of sparsely distributed trees and bushes.

[ece33824-bib-0025] Kjær, E. D. , Lobo, A. , & Myking, T. (2014). The role of exotic tree species in Nordic forestry. Scandinavian Journal of Forest Research, 29, 323–332. https://doi.org/10.1080/02827581.2014.926098

[ece33824-bib-0026] Kjær, E. D. , McKinney, L. V. , Nielsen, L. R. , Hansen, L. N. , & Hansen, J. K. (2012). Adaptive potential of ash (*Fraxinus excelsior*) populations against the novel emerging pathogen *Hymenoscyphus pseudoalbidus* . Evolutionary Applications, 5, 219–228. https://doi.org/10.1111/j.1752-4571.2011.00222.x 25568043 10.1111/j.1752-4571.2011.00222.x PMC3353348

[ece33824-bib-0027] Kremer, A. , Ronce, O. I. , Robledo‐Arnuncio, J. J. , Guillaume, F. D. R. , Bohrer, G. , Nathan, R. , … Schueler, S. (2012). Long‐distance gene flow and adaptation of forest trees to rapid climate change. Ecology Letters, 15, 378–392. https://doi.org/10.1111/j.1461-0248.2012.01746.x 22372546 10.1111/j.1461-0248.2012.01746.x PMC3490371

[ece33824-bib-0028] Krüsi, B. O. , & Debussche, M. (1988). The fate of flowers and fruits of *Cornus sanguinea* L. in three contrasting Mediterranean habitats. Oecologia, 74, 592–599. https://doi.org/10.1007/BF00380058 28311767 10.1007/BF00380058

[ece33824-bib-0029] Kubisch, A. , Holt, R. D. , Poethke, H. J. , & Fronhofer, E. A. (2014). Where am I and why? Synthesizing range biology and the eco‐evolutionary dynamics of dispersal. Oikos, 123, 5–22. https://doi.org/10.1111/j.1600-0706.2013.00706.x

[ece33824-bib-0030] Larsen, J. B. (1986). Silver fir decline: A new hypothesis concerning this complex decline syndrome in *Abies alba* (Mill.). Forstwissenschaftliches Centralblatt, 105, 381–396. https://doi.org/10.1007/BF02741747

[ece33824-bib-0031] Larsen, A. S. , Asmussen, C. B. , Coart, E. , Olrik, D. C. , & Kjær, E. D. (2006). Hybridization and genetic variation in Danish populations of European crab apple (*Malus sylvestris*). Tree Genetics & Genomes, 2, 86–97. https://doi.org/10.1007/s11295-005-0030-0

[ece33824-bib-0032] Larsen, A. S. , & Kjær, E. D. (2009). Pollen mediated gene flow in a native population *of Malus sylvestris* and its implications for contemporary gene conservation management. Conservation Genetics, 10, 1637 https://doi.org/10.1007/s10592-008-9713-z

[ece33824-bib-0033] Lascoux, M. , Glémin, S. , & Savolainen, O. (2016). Local adaptation in plants. eLS. Chichester, UK: John Wiley & Sons, Ltd https://doi.org/10.1002/9780470015902.a0025270

[ece33824-bib-0034] Menzel, A. , & Fabian, P. (1999). Growing season extended in Europe. Nature, 397, 659 https://doi.org/10.1038/17709

[ece33824-bib-0035] Menzel, A. , Sparks, T. H. , Estrella, N. , Koch, E. , Aasa, A. , Ahas, R. , … Zust, A. (2006). European phenological response to climate change matches the warming pattern. Global Change Biology, 12, 1969–1976. https://doi.org/10.1111/j.1365-2486.2006.01193.x

[ece33824-bib-0036] Myking, T. , Rusanen, M. , Steffenrem, A. , Kjær, E. D. , & Jansson, G. (2016). Historic transfer of forest reproductive material in the Nordic region: Drivers, scale and implications. Forestry, 89, 325–337. https://doi.org/10.1093/forestry/cpw020

[ece33824-bib-0037] Nadeau, S. , Meirmans, P. G. , Aitken, S. N. , Ritland, K. , & Isabel, N. (2016). The challenge of separating signatures of local adaptation from those of isolation by distance and colonization history: The case of two white pines. Ecology and Evolution, 6, 8649–8664. https://doi.org/10.1002/ece3.2550 28035257 10.1002/ece3.2550 PMC5192886

[ece33824-bib-0038] Nicotra, A. B. , Atkin, O. K. , Bonser, S. P. , Davidson, A. M. , Finnegan, E. J. , Mathesius, U. , … van Kleunen, M. (2010). Plant phenotypic plasticity in a changing climate. Trends in Plant Science, 15, 684–692. https://doi.org/10.1016/j.tplants.2010.09.008 20970368 10.1016/j.tplants.2010.09.008

[ece33824-bib-0039] Nikulin, G. , Kjellström, E. , Hansson, U. , Strandberg, G. , & Ullerstig, A. (2011). Evaluation and future projections of temperature, precipitation and wind extremes over Europe in an ensemble of regional climate simulations. Tellus A, 63, 41–55. https://doi.org/10.1111/j.1600-0870.2010.00466.x

[ece33824-bib-0040] Ødum, S. (1968). Udbredelsen af træer og buske i Danmark. Botanisk Tidskrift, 64, 1–118.

[ece33824-bib-0041] Petit, R. J. , & Hampe, A. (2006). Some evolutionary consequences of being a tree. Annual Review of Ecology Evolution and Systematics, 37, 187–214. https://doi.org/10.1146/annurev.ecolsys.37.091305.110215

[ece33824-bib-0042] Pluess, A. R. , Frank, A. , Heiri, C. , Lalagüe, H. , Vendramin, G. G. , & Oddou‐Muratorio, S. (2016). Genome‐environment association study suggests local adaptation to climate at the regional scale in *Fagus sylvatica* . New Phytologist, 210, 589–601. https://doi.org/10.1111/nph.13809 26777878 10.1111/nph.13809

[ece33824-bib-0043] Rehfeldt, G. E. , Ying, C. C. , Spittlehouse, D. L. , & Hamilton, D. A. (1999). Genetic responses to climate in *Pinus contorta*: Niche breadth, climate change and reforestation. Ecological Monographs, 69, 375–407. https://doi.org/10.1890/0012-9615(1999)069[0375:GRTCIP]2.0.CO;2

[ece33824-bib-0044] Reim, S. , Proft, A. , Heinz, S. , Lochschmidt, F. , Höfer, M. , Tröber, U. , & Wolf, H. (2015). Pollen movement in a *Malus sylvestris* population and conclusions for conservation measures. Plant Genetic Resources, 15, 1–9. https://doi.org/10.1017/S1479262115000301

[ece33824-bib-0045] Richardson, A. D. , Andy Black, T. , Ciais, P. , Delbart, N. , Friedl, M. A. , Gobron, N. , … Varlagin, A. (2010). Influence of spring and autumn phenological transitions on forest ecosystem productivity. Philosophical Transactions of the Royal Society of London B: Biological Sciences, 365, 3227–3246. https://doi.org/10.1098/rstb.2010.0102 20819815 10.1098/rstb.2010.0102 PMC2981939

[ece33824-bib-0046] Richardson, A. D. , Keenan, T. F. , Migliavacca, M. , Ryu, Y. , Sonnentag, O. , & Toomey, M. (2013). Climate change, phenology, and phenological control of vegetation feedbacks to the climate system. Agricultural and Forest Meteorology, 169, 156–173. https://doi.org/10.1016/j.agrformet.2012.09.012

[ece33824-bib-0047] Roberts, A. V. , Gladis, T. , & Brumme, H. (2009). DNA amounts of roses (*Rosa* L.) and their use in attributing ploidy levels. Plant Cell Reports, 28, 61–71. https://doi.org/10.1007/s00299-008-0615-9 18839177 10.1007/s00299-008-0615-9

[ece33824-bib-0048] Rohde, A. , Bastien, C. , & Boerjan, W. (2011). Temperature signals contribute to the timing of photoperiodic growth cessation and bud set in poplar. Tree Physiology, 31, 472–482. https://doi.org/10.1093/treephys/tpr038 21636689 10.1093/treephys/tpr038

[ece33824-bib-0049] Salmela, M. J. , Cavers, S. , Cottrell, J. E. , Iason, G. R. , & Ennos, R. A. (2013). Spring phenology shows genetic variation among and within populations in seedlings of Scots pine (*Pinus sylvestris* L.) in the Scottish Highlands. Plant Ecology & Diversity, 6, 523–536. https://doi.org/10.1080/17550874.2013.795627

[ece33824-bib-0050] SAS Institute Inc. (2011). SAS/STAT^®^ 9.3 user's guide. Cary, NC: SAS Institute Inc..

[ece33824-bib-0051] Satterthwaite, F. E. (1946). An approximate distribution of estimates of variance components. Biometrics Bulletin, 2, 110–114. https://doi.org/10.2307/3002019 20287815

[ece33824-bib-0052] Savolainen, O. , Pyhäjärvi, T. , & Knürr, T. (2007). Gene flow and local adaptation in trees. Annual Review of Ecology Evolution and Systematics, 38, 595–619. https://doi.org/10.1146/annurev.ecolsys.38.091206.095646

[ece33824-bib-0053] Smith, S. A. , & Donoghue, M. J. (2008). Rates of molecular evolution are linked to life history in flowering plants. Science, 322, 86 https://doi.org/10.1126/science.1163197 18832643 10.1126/science.1163197

[ece33824-bib-0054] Sork, V. L. (2016). Gene flow and natural selection shape spatial patterns of genes in tree populations: Implications for evolutionary processes and applications. Evolutionary Applications, 9, 291–310. https://doi.org/10.1111/eva.12316 27087853 10.1111/eva.12316 PMC4780383

[ece33824-bib-0055] Spitze, K. (1993). Population structure in *Daphnia obtusa*: Quantitative genetic and allozymic variation. Genetics, 135, 367–374. 8244001 10.1093/genetics/135.2.367 PMC1205642

[ece33824-bib-0056] Stanturf, J. A. , Kant, P. , Lillesø, J. P. B. , Mansourian, S. , Kleine, M. , Graudal, L. , & Madsen, P. (2015). Forest landscape restoration as a key component of climate change mitigation and adaptation. Vienna, Austria: International Union of Forest Research Organizations (IUFRO).

[ece33824-bib-0057] Valladares, F. , Matesanz, S. , Guilhaumon, F. , Araújo, M. B. , Balaguer, L. , Benito‐Garzón, M. , … Naya, D. E. (2014). The effects of phenotypic plasticity and local adaptation on forecasts of species range shifts under climate change. Ecology Letters, 17, 1351–1364. https://doi.org/10.1111/ele.12348 25205436 10.1111/ele.12348

[ece33824-bib-0058] Vitasse, Y. , Delzon, S. , Bresson, C. C. , Michalet, R. , & Kremer, A. (2009). Altitudinal differentiation in growth and phenology among populations of temperate‐zone tree species growing in a common garden. Canadian Journal of Forest Research, 39, 1259–1269. https://doi.org/10.1139/X09-054

[ece33824-bib-0059] Vitasse, Y. , Delzon, S. , Dufrêne, E. , Pontailler, J. Y. , Louvet, J. M. , Kremer, A. , & Michalet, R. (2009). Leaf phenology sensitivity to temperature in European trees: Do within‐species populations exhibit similar responses?. Agricultural and Forest Meteorology, 149, 735–744. https://doi.org/10.1016/j.agrformet.2008.10.019

[ece33824-bib-0060] Way, D. A. (2011). Tree phenology responses to warming: Spring forward, fall back?. Tree Physiology, 31, 469–471. https://doi.org/10.1093/treephys/tpr044 21636688 10.1093/treephys/tpr044

