# Supplementary material for: Differences among six woody perennials native to Northern Europe in their level of genetic differentiation and adaptive potential at fine local scale
Source: Ecol Evol. 2018 Jan 25;8(4):2231–9. doi: 10.1002/ece3.3824 (PMC5817134; doi:10.1002/ece3.3824)
Supplement: Supplementary file 1 [file ECE3-8-2231-s001.pdf]

Description of the species field trials, location of populations and climate information at the population site

1. ***Betula pubescens* : Downy birch**
   Trial # F413- Himmerige Skove, Hinnerup
   Location: 56.24 latitude, 10.10 longitude
   Area: 0.9 ha
   Planting year: 2007
   Planting design: 12 blocks with 40 family plots of 4 trees per plot per block
   Planting distance: 3x1 m

#	Populations	Latitude	Longitude	No:of families	T*min* March(^{0}C)
1	Buskene	56.28	8.95	11	-1.1
2	Gjern Bakker	56.22	9.72	13	-2.5
3	Hald Ege	56.38	9.33	4	-1.8
4	Kongens Mose	55.00	8.97	15	-0.1
5	Pøtsø Mose	56.15	9.50	14	-1.9
6	Svanemosen	55.42	9.42	8	-0.9
7	Tofte Skov	56.83	10.22	9	-1.7
8	Aaby Mose	57.18	9.75	14	-1.2

2. ***Cornus sanguinea*: Common dogwood**
   Trial # F421- Drastrup Skov, Himmerland
   Location: 56.99 latitude, 9.83 longitude
   Area: 0.5 ha
   Planting year: 2008
   Planting design: 7 blocks with 80 family plots of 3 trees in each block (240 trees/block)
   Planting distance: 3x1 m

#	Populations	Latitude	Longitude	No:of families	T*min* May(^{0}C)
1	Sebbelev skov	54.96	9.84	2	7.4
2	Skodsbøl skov	54.91	9.63	14	7.3
3	Himmerland	56.88	9.88	1	7.4
4	Sønder kongerslev	56.88	10.13	17	7.3
5	Vive kyst	56.70	10.05	3	7.5
6	Stenderup	55.47	9.65	6	7.2
7	Båring skov	55.50	9.92	4	7.2
8	Kirstinebjerg	55.60	9.78	9	7.3
9	Selvejerskov	55.60	9.82	8	7.4
10	Borum skov	56.18	9.98	3	7.6

11	Grund skov	55.69	9.75	1	7.2
12	Stouby skov	55.68	9.80	3	7.3
13	Lodskov	55.30	10.57	1	7.1
14	V. Skerninge	55.05	10.47	1	7.3
15	Købelev skov	54.93	11.08	1	7.4
16	Stege nord F	54.97	12.30	1	7.7
17	Vesterlyng	55.73	11.28	1	8.2
18	Allindelille	55.52	11.75	1	7.9

3. Corylus avellana: Common hazel

Trial # F446- Anebjerg Skov, Skanderborg
Location: 56.30 latitude, 9.59 longitude
Area: 1.06 ha
Planting year: 2011
Planting design: 10 blocks with 4 rows of 22 family plots per row and 4 trees per plot
Planting distance: 3x1 m

#	Populations	Latitude	Longitude	No:of families	T*min* March(^{0}C)
1	Damgård Skov	55.90	9.39	8	-1.8
2	Langå Egeskov	56.38	9.90	4	-2.2
3	Mausing Skov	56.24	9.45	3	-2.0
4	Vands Skov	56.21	9.40	2	-1.9
5	Kabbel Krat	56.56	8.31	2	-0.5
6	Åmølle Krat	56.54	8.49	4	-0.6
7	Strandbjerg Krat	56.52	8.58	2	-0.7
8	Fosdalen	57.13	9.40	5	-1.7
9	Skovsgård Krat	57.11	9.49	3	-1.5
10	Dyrehaven Frijsenborg	56.27	9.88	5	-2.5
11	Staurhug	56.24	9.27	2	-1.7
12	Skovsagergård Krat	57.50	10.01	1	-0.9
13	Bjergby Krat	57.51	10.04	2	-1.0
14	Grimmeshave Krat	57.49	10.07	2	-1.0
15	Nørbjerg	57.19	9.75	1	-1.2
16	Skeelslund Skov	57.12	9.74	2	-1.3
17	Ulveskov	57.13	9.64	5	-1.3
18	Højris Plantage Kystkrat	56.74	8.82	4	-0.9
19	Brigsbjerg Krat	56.61	8.69	1	-0.7
20	Kås Skov	56.62	8.70	5	-0.8
21	Lindholt Krat Sevel	56.44	8.82	2	-1.3
22	Sevel Krat	56.46	8.86	2	-1.1
23	Skivum Nørrekrat	56.87	9.59	11	-2.2

24	Hesselkrattene	56.68	9.16	4	-1.5
25	Junget Skov	56.77	9.11	7	-1.5
26	Jenle Krat	56.66	9.08	10	-1.5

4. *Malus sylvestris*: European crab apple

Trial # F392- Soldaterskoven, Lindet Statsskovdistrikt
Location: 54.96 latitude, 8.85 longitude
Area: 0.9 ha
Planting year: 2005
Planting design: 6 randomized blocks with 10 rows per block. Each row has 49 plants distributed in half sib family plots of 5 trees per plot (One plot in each row had only 4 trees).
Planting distance: 3x1 m

#	Populations	Latitude	Longitude	No:of families	T*min* May(^{0}C)
1	Ajstrup	56.70	10.22	17	7.4
2	Boserup	55.66	12.04	9	7.9
3	Daugbjerg	56.45	9.14	5	7
4	Draved	55.02	8.97	1	6.8
5	Gulstav	54.72	10.72	6	7.2
6	Gurre V	56.02	12.48	13	8
7	Kosteskov	54.78	11.69	17	7.8
8	Lovnkaer	56.72	10.20	8	7.4
9	Lundbaek	56.96	9.60	3	7.6
10	Nørbjerg	57.19	9.76	4	7
11	Oplev	56.84	9.81	2	7.4
12	Rinkenaes	54.92	9.55	3	6.8

5. *Quercus petraea*: Sessile oak

Trial # F373- Randers Fælled
Location: 56.49 latitude, 10.02 longitude
Area: 5 ha
Planting year: 2002
Planting design: 45 blocks, 9 rows per block, each row has 6 half sib family plots of 4 trees each
Planting distance: 3x1 m

#	Populations	Latitude	Longitude	No:of families	*Tmin* May(^{0}C)
1	Fussingø	56.45	9.83	10	7.6
2	Hald ege	56.39	9.34	8	7.3
3	Linnebjerg	56.20	9.11	13	6.6

4	Løvbakke	56.18	8.94	13	6.6
5	Lysbro	56.16	9.51	4	7.4
6	Tværskov	56.11	9.41	9	6.9
7	Hørbylunde	56.13	9.40	12	6.9
8	Dejbjerg	55.99	8.43	14	7
9	Skarrild	55.99	8.91	4	6.6
10	Johanne Plantage	55.94	9.26	3	6.7
11	Tinnet	55.90	9.37	4	6.6
12	Løndal Næs	56.07	9.60	5	7.4
13	Stenholt Klosterlund	56.18	9.35	4	6.9
14	Buderupholm	56.81	9.87	3	7.2
15	Tved skov	56.81	9.85	3	7.2
16	Hønning	55.19	8.95	7	6.6
17	Lovrup	55.14	8.88	1	6.9
18	Løndal	56.03	9.58	8	7.1
19	Silkeborg Nordskov	56.15	9.60	7	7.5

6. *Rosa dumalis*: Glaucous dog rose

Trial # F422- Drastrup Skov, Himmerland

Location: 56.59 latitude, 9.49 longitude

Area: 0.2 ha

Planting year: 2008

Planting design: 5 randomized blocks, with family plots of 3 plants each, arranged in 32 rows, with 18 plots per row.

Planting distance: 3x1 m

#	Populations	Latitude	Longitude	No:of families	*Tmin* May(^{0}C)
**1**	Glatved Strand	56.31	10.87	13	7.7
**2**	Jernhatten	56.25	10.79	8	7.4
**3**	Lushage, Helgenæs	56.10	10.54	5	7
**4**	Røjen, Skødshoved	56.20	10.38	1	7
**5**	Ildal Bæk, Salten	56.09	9.60	6	7.3
**6**	Tannebæk Dal	56.56	8.31	6	7.2
**7**	Gedebjerg	57.35	10.47	4	7.2
**8**	West of Øksnebjerg	57.39	10.47	3	7.1
**9**	Skagens Gren	57.74	10.64	2	7.3
**10**	Kandestederne	57.66	10.39	2	7.2
**11**	Råbjerg Stene	57.64	10.35	1	7.1
**12**	Skiveren	57.62	10.28	3	7.1
**13**	Volsted	56.89	9.91	2	7.2
**14**	Mulbjerge	56.91	10.26	6	7.3
**15**	Fladbjerg	56.67	10.01	2	7.5
**16**	Febbersted Kløft, Hanstholm	57.11	8.68	4	6.9

**17**	Hegedal	56.65	9.83	2	7.7
**18**	Færgehagen Strand, Mariager	56.66	9.97	3	7.6
**19**	Lundshøj,Kielstrup Sø	56.66	9.93	2	7.5
**20**	Låenhus Eneoverdrev	56.66	9.94	3	7.5
**21**	Holbæk Kær, Stinesminde	56.68	9.99	3	7.6
**22**	Lysnet Bakke	56.37	9.96	7	7.4
**23**	Boddum Bakker	56.70	8.46	3	7.1
**24**	Jørsby, Buksør Odde	56.88	8.90	8	7.2
**25**	Vesterklit, Tversted	57.60	10.22	2	7.1
**26**	Brudehøj,Bjergby	57.53	10.05	1	6.8
**27**	Tornby Klitplantage	57.54	9.90	5	6.9
**28**	Tornby Strand	57.55	9.91	4	6.9
**29**	Skallerup Indlandsklitter	57.49	9.86	2	6.9
**30**	Sanden,Lien	57.14	9.37	2	7.4
**31**	Øster Hegnet	56.72	9.16	2	7.5
